# VALIDATION OF A COMBINED SARCOPENIA AND NUTRITION SCREENING TOOL FOR COMMUNITY-LIVING OLDER ADULTS

**DOI:** 10.1093/geroni/igae098.2835

**Published:** 2024-12-31

**Authors:** Susan Saffel-Shrier, Amy Covington, Jing Wang

**Affiliations:** University of Utah, Salt Lake City, Utah, United States; University of Utah, Salt Lake City, Utah, United States; University of Utah, Salt Lake City, Utah, United States

## Abstract

Malnutrition and sarcopenia are major intersecting conditions of the frailty syndrome. Both have similar symptoms to include reduced energy and protein intake along with losses of muscle and strength. These losses can lead to decreased functionality and independence culminating in poor health outcomes. When both conditions are present, community-living older adults have a potential for a more direct negative health effect. Appropriate screening tools for malnutrition and sarcopenia are frequently underutilized impairing diagnosis and treatment in this population. Furthermore, there is a lack of validated community-based nutrition screening tools compared against the Nutrition-Focused Physical Exam (NFPE), the gold standard for malnutrition diagnosis. The purpose of this study was to determine if combining two screening tools, the nutrition SCREEN II and sarcopenia SARC-F would yield higher malnutrition diagnostic accuracy compared to using each tool individually. Utilizing the NFPE tool as a reference, the combined SCREEN II plus SARC-F accurately diagnosed malnutrition in 103 participants (84.4%), surpassing the individual diagnoses of 72 (59%) by SCREEN II and 66 (54%) by SARC-F. This result underscores the superior accuracy of the combined tool. The internal consistency reliability of the combined tool, assessed through Cronbach’s alpha, yielded a coefficient of 0.73, indicating satisfactory internal consistency reliability. The convergent validity, assessed using the Phi coefficient, was 0.67, suggesting that the combined scores from the two tools are positively correlated with diagnoses of malnutrition based on the NFPE. These results support utilization of the combined screening tools in diagnosing malnutrition therefore improving the health of older adults.

